# Gamma knife radiotherapy in a neurofibromatosis type 1 Chinese pedigrees with *NF1* gene frameshift mutation: A case report

**DOI:** 10.1097/MD.0000000000029280

**Published:** 2022-07-08

**Authors:** Meng-Jie Dong, Zhong-Kun Yang, Ji Yang, Rui-Qin Guo, Yu-Yuan Xiao, Hai Liu

**Affiliations:** a Department of Ophthalmology, The Affiliated Hospital of Yunnan University, The Second People’s Hospital of Yunnan Province, Kunming, China; b The Eye Disease Clinical Medical Research Center of Yunnan Province, Kunming, China; c The Eye Disease Clinical Medical Center of Yunnan Province, Kunming, China.

**Keywords:** frameshift mutations, gamma knife radiotherapy, optic glioma, neurofibromatosis type 1, *NF1* gene

## Abstract

**Rationale:**

Neurofibromatosis type 1 (NF1) is a common autosomal dominant genetic disorder. NF1 is a multisystemic disease and its pathogenesis involves mutations in the *NF1* gene on chromosome 17q11.2 causing RAS overactivation to stimulate abnormal cell proliferation. In this article, a Chinese family with neurofibromatosis type 1 was reported and the relationship between the phenotype and gene mutation was analyzed.

**Patient concerns:**

The patient was a 9-year-old-male child diagnosed with right eye exophthalmos combined with right eye glioma, optic edema, and peripheral visual field defect. There were multiple cafe-au-lait spots in the whole body of the child. His mother had multiple cafe-au-lait spots, and the eye examination showed no abnormalities.

**Diagnosis:**

The proband was diagnosed with NF1 and a heterozygous frameshift mutation (c. 6641delG p. Arg2214Asnfs*30) in the *NF1* gene was identified, and his mother also carried the same pathogenic mutation.

**Interventions:**

To protect the vision of the right eye, he was treated with gamma knife radiotherapy.

**Outcomes:**

After therapy, his fundus optic disc edema was decreased and the best corrected visual acuity of the right eye was increased.

**Lessons:**

Gene detection is helpful to diagnose the disease and guide the treatment. Gamma knife radiotherapy can preserve better neurological function.

## 1. Introduction

Neurofibromatosis type 1 is an autosomal dominant disease caused by *NF1* gene mutation. *NF1* gene is located on chromosome 17q11.2, the size is about 350 kb, containing 58 exons, encoding 2818 amino acids.^[1–3]^ As a negative feedback regulator of RAS signaling pathway, *NF1* gene is responsible for encoding nerve fiber protein, which mainly exists in glial cells, neurons and Schwann cells. *NF1* gene mutation and functional disorder lead to the loss of nerve fiber protein function, which lead to RAS overactivated, thus stimulating cell proliferation and leading to a various of tumors occurrence.^[4,5]^ The malignant change rate of tumors is about 7%.^[6,7]^

Neurofibromatosis type 1 is a monogenic disease caused by mutations in the *NF1* gene. The *NF1* gene is the only definite pathogenic gene. In this study, the traditional sanger sequencing method was used to identify the pathogenic mutations of the *NF1* gene.^[8]^ Clinical phenotypes might vary in patient with neurofibromatosis type 1, but individuals in this study were diagnosed with NF1 only when they met 2 or more of the neurofibromatosis type 1 diagnostic criteria of the National Institutes of Health.^[9]^ In this article, we reported the clinical phenotype and genetic characteristics of patients in a family with neurofibromatosis type 1 confirmed by clinical symptoms and gene detection.

## 2. Case report

**Figure 1.**
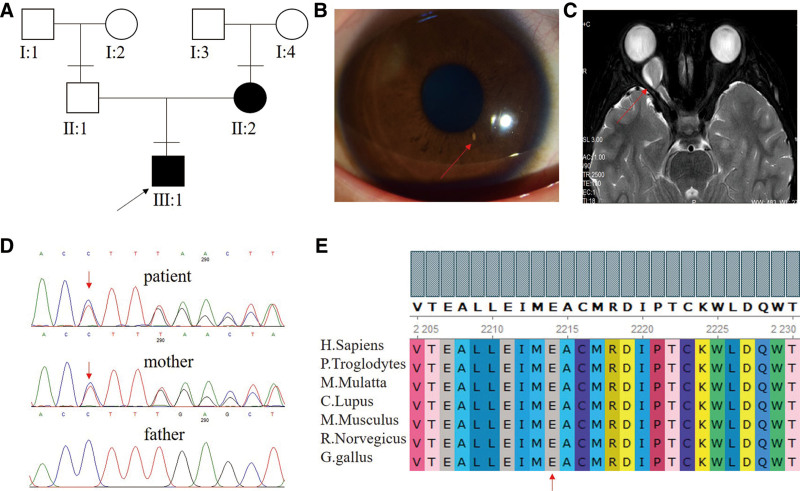
Clinical information. (A) Males are denoted with squares and females with circles. Empty and filled symbols indicate unaffected and affected individuals. Arrow represents the proband. (B) The proband has a Lisch nodule change in his right iris. (C) MRI reveals the formation of optic glioma in the proband’s right eye. (D) A heterozygous c.6641delG mutation in exon 43 of *NF1* gene in proband was found. The mother of the patient carried the same pathogenic mutation. The healthy father did not carry the pathogenic mutation. (E) Conservative analysis showed that amino acid at 2214 site of *NF1* gene was highly conserved among multimodal organisms.

**Figure 2.**
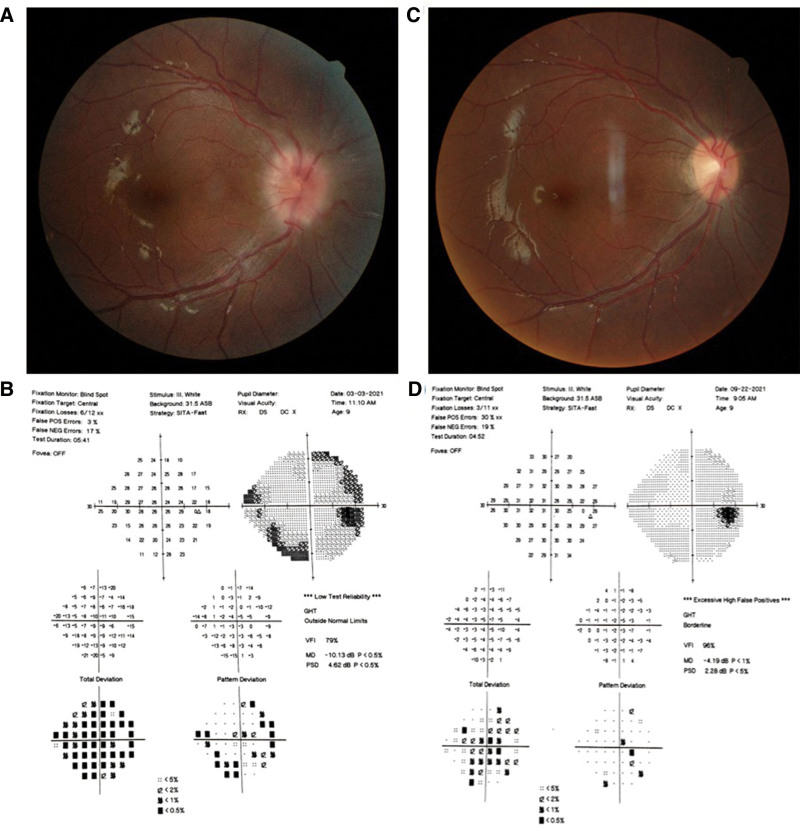
Pretherapy and posttreatment: fundus and visual field clinical examination of proband. (A) The optic disc in the right fundus of the proband has obvious edema. (B) Visual field examination reveals a peripheral visual field defect of the proband’s right eye. (C) Six months after the operation, the optic disc edema decreased. (D) The peripheral visual field defect was better than that before the operation.

Proband was a 9-year-old-male child diagnosed with right eye exophthalmos (Fig. 1A). The naked vision was right eye 0.4, left eye 1.0, and the correction of right eye vision did not improve. The slit lamp examination showed that there was no obvious abnormality in the right eye cornea of the child. Multiple Lisch nodules were seen in the iris (Fig. 1B). The edema of the right eye optic disc was obvious (Fig. 2A). The visual field examination showed the peripheral visual field defect (Fig. 2B). At the same time, there were multiple cafe-au-lait spots in the whole body of the child. MRI examination showed the right eye glioma (Fig. 1C). The patient underwent resection of the left lumbar neurofibroma at age 4. The mother had multiple cafe-au-lait spots, and the eye examination showed no abnormalities. There was no abnormality in father examination. We found a heterozygous c.6641delG mutation in exon 43 of the *NF1* gene in proband (Fig 1D). The sicked mother of the patient also carried the same pathogenic mutation (Fig 1D), which resulted in premature termination of neurofibroma protein translation at amino acid 2244 (p. Arg2214Asnfs*30), and the healthy father did not carry the pathogenic mutation (Fig 1D). This mutation has been reported in Clinvar database.^[10]^ Conservative analysis showed that amino acid at 2214 *NF1* gene was highly conserved among multimodal organisms (Fig.1E). The right eye optic glioma of the proband affected the optic nerve. His right eye vision began to decline, and the optic disc was compressed and swollen. We decided to protect the vision of the right eye as much as possible. After the family members of the patients were informed of the disease and the feasible treatment plan in detail, the family members of the patients unanimously decided to accept gamma knife radiotherapy. To reduce the negative impact of radiation on optic chiasma, we divided the treatment for 3 times, the respective doses were 5.5, 5.5, and 5.0 Gy marginal dose. Half year after operation, the best corrected visual acuity of the right eye of patient increased to 0.8 (0.4 before operation), the fundus optic disc edema also decreased, and the peripheral visual field defect was improved (Fig. 2C and D).

## 3. Discussion

Neurofibroma type 1 is a common autosomal dominant genetic disorder with obvious clinical and genetic heterogeneity. There are different clinical symptoms between different patients, and the same patient performs differently at different ages. For example, the proband had no obvious symptoms at birth, and gradually appeared cafe-au-lait spots, optic glioma and neurofibroma in childhood. Even in the same family, the clinical manifestations of different patients were also very different. For example, the proband’s MRI examination found optic glioma, while the mother with the same pathogenic mutation had no such phenotype.

At present, the best treatment for optic nerve glioma in patients with neurofibroma type 1 is still controversial. The timing of treatment depends primarily on the presence of progressive visual loss, not necessarily on tumor growth observed on imaging.^[11]^ The treatment of optic glioma in children is still a great challenge. There is growing evidence that gamma knife therapy can control tumor growth.^[12]^ Gamma knife surgery preserves better neurological function and has fewer treatment-related complications over long-term follow-up.^[13]^ We can increase the sensitivity of tumor tissues to radiation by adjusting the dose of each treatment.^[14,15]^ The treatment of optic glioma needs individualized methods. Based on genetic detecting result, the proband was treated with a gamma knife. Half year after operation, the best corrected visual acuity of the right eye of patient was increased and the fundus optic disc edema also decreased, dramatically the peripheral visual field defect was also improved. Therefore, we think gamma knife therapy has well curative effect for proband.

Neurofibromatosis type 1 (NF1) is an autosomal dominant hereditary disease. Homozygous mutations are lethal to the embryo, so the affected individuals are heterozygous mutations in the *NF1* gene.^[16,17]^ In this study, the proband and his sick mother with clinical manifestations showed heterozygous mutations. Patients with frameshift mutations are reported to be more prone to suffer from Lisch nodules and neurofibromas.^[18]^ In this study, the proband with *NF1* gene frameshift mutations confirmed by genetic testing showed significant Lisch nodules and neurofibromas. *NF1* gene mutation types are diverse, and most mutations are nonsense mutations.^[7]^ So far, >3000 different *NF1* gene mutations have been identified in the human gene mutation database. But more research is needed to analyze the association between clinical phenotypes and gene mutation types.^[19–21]^ In summary, we detected the *NF1* gene pathogenic mutation in NF1 patients, and further clarified the diagnosis at the gene level, which laid the foundation for the prenatal diagnosis, genetic counseling and analysis of the correlation between genotype and phenotype.^[22,23]^

## Author contributions

H.L. was involved in conceptualization and resources. J.Y. was involved in data curation. R.-Q.G. and Y.-Y.X. were involved in formal analysis. Z.K.Y. and M.J.D. were involved in investigation. Z.-K.Y. and H.L. were involved in methodology.M.-J.D. and J.Y. were involved in writing—original draft.
